# Bioactive Assessment of MMA-Based Dental Materials: Molecular Docking and Network Topology Analysis of Stress-Regulated Survival, Apoptosis, and Mechanotransduction Pathways

**DOI:** 10.3390/cimb48060630

**Published:** 2026-06-17

**Authors:** Yağmur Dilber, Erhan Dilber, Kübra Yıldız Domaniç

**Affiliations:** 1 Department of Medical Biochemistry, Faculty of Medicine, Akdeniz University, Antalya 07070, Türkiye; yagmursarica88@gmail.com; 2 Department of Prosthodontics, Faculty of Dentistry, Alanya Alaaddin Keykubat University, Alanya 07490, Türkiye; erhan.dilber@alanya.edu.tr; 3 Department of Prosthodontics, Faculty of Dentistry, Atlas University, İstanbul 34408, Türkiye

**Keywords:** methyl methacrylate, in silico biocompatibility, molecular docking, osseointegration, ferroptosis, PPI network analysis

## Abstract

Methyl methacrylate (MMA)-based materials are widely used in temporary and permanent prosthetic dentistry; the prolonged presence of these materials in the oral cavity and potential residual monomer release can affect local biological responses. This study aimed to evaluate the biocompatibility and toxicity profiles of MMA, the monomeric unit of polymethyl methacrylate (PMMA), a key component of dental materials used in temporary prosthetic restorations. Molecular docking simulations were performed using CB-Dock2 and Autodock vina, while protein–protein interaction (PPI) analysis was performed using STRING and Cytoscape. In addition, Swiss ADME Target Prediction, toxicity prediction, and enrichment analyses were used to characterize the biological significance of selected targets in more detail. Molecular docking studies revealed promising interactions of MMA with valuable biomolecular targets relevant to biocompatibility. The toxicity profile revealed aspects of MMA that could be improved. Pharmacophore modeling, highlighting the importance of carbonyl and hydroxyl groups as pharmacophoric properties, revealed compounds with suitable biocompatibility profiles. Consequently, it emphasizes the interactions of MMA with biomolecules and safety considerations. It can guide the design and optimization of biocompatible materials as an exploratory avenue for future developments in dental biomaterials.

## 1. Introduction

Temporary prosthetic restorations constitute a vital component of contemporary dental practice, offering temporary yet functional solutions until permanent prostheses can be applied to replace missing or damaged teeth. These restorations represent a critical transitional phase in dental treatment, providing patients with immediate esthetic and functional rehabilitation while long-term treatment procedures are being completed [1,2]. Beyond their mechanical roles, these restorations significantly contribute to the preservation of facial appearance and oral fit, which are closely linked to patient comfort, confidence, and psychosocial well-being [3,4,5,6].

PMMA, a synthetic polymer derived from MMA monomer, remains one of the most used materials in the fabrication of temporary prosthetic restorations and forms a fundamental element of prosthetic workflows. PMMA has gained widespread acceptance in dentistry due to its suitability for short-term intraoral applications, owing to its favorable properties, such as acceptable biocompatibility, relatively low cytotoxicity and ease of clinical manipulation [7,8,9]. Clinical experience has shown that PMMA-based materials typically produce limited irritation or inflammatory responses in oral tissues and can therefore be used during temporary treatment periods without causing significant disruption to surrounding soft or hard tissues [10,11]. Although PMMA-based materials have long been regarded as clinically useful and relatively inert, the expanding use of 3D-printing technologies in dentistry has renewed attention to their biological behavior, particularly with regard to biocompatibility and toxicity [12,13,14,15,16,17].

Driven by concerns over residual monomer release and resin-associated cytotoxicity, bio-based resin formulations are increasingly being investigated as promising strategies to improve the biological safety of conventional dental resin systems [18].

Biocompatibility remains a critical consideration for all dental materials, as it reflects a material’s ability to function without generating adverse local or systemic biological responses. In the context of temporary restorations, the biological performance of gingival-mediated PMMA is influenced by numerous factors, including residual monomer release, surface properties, and the nature of its interactions with living tissues, all of which can collectively affect cellular behavior and tissue response [19]. Although PMMA is generally considered to exhibit low toxicity, accumulating evidence suggests that residual monomers and material-related eluates can disrupt cellular redox homeostasis and trigger stress-related cytotoxic responses, including mitochondrial apoptosis [20]. In particular, dental methacrylate monomers have been reported to increase reactive oxygen species (ROS) levels and trigger antioxidant defense responses involving the Nrf2 pathway, underscoring the relevance of redox-regulated signaling in the evaluation of material biocompatibility [21,22].

Ideal properties for PMMA-based materials include minimizing residual monomer-related biological stress, supporting cellular homeostasis and stress-adaptive survival signaling in intraoral tissues, and limiting adverse pathway perturbations that may indirectly affect tissue healing and integration. In this context, it has been suggested that the phosphatase and tensin homolog (PTEN)/protein kinase B, PKB (AKT)/Mammalian Target of Rapamycin (mTOR)/hypoxia-inducible factor-1(HIF1A) signaling pathway plays a role in regulating osteogenic differentiation, metabolic adaptation, and bone formation around dental implants and is critical in implant-associated tissue integration [23,24,25,26,27]. Conversely, sustained redox imbalance can shift cellular fate toward apoptosis through canonical mediators such as B-cell lymphoma 2(BCL-2) family signaling and CASPASE-3 execution, providing a mechanistically consistent link between oxidative stress responses and impaired cellular viability [28,29,30]. Yes-associated protein and its transcriptional coactivator with PDZ-binding motif (YAP/TAZ) are two homologous transcriptional coactivators that lie at the center of a potential regulatory network of Hippo, Wnt, GPCR, estrogen, mechanical, and metabolism signaling. YAP/TAZ influences the expressions of downstream genes and proteins, as well as enzyme activity in metabolic cycles, cell proliferation, inflammatory factor expression, and the transdifferentiation of fibroblasts into myofibroblasts [31]. In this study, we systematically investigated the interactions of MMA, the base monomer of PMMA, with key signaling proteins using in silico approaches, leveraging the power of computer-based environments to examine mechanisms at the molecular level. The primary aim was to characterize the potential binding sites and relative interaction tendencies of MMA-associated complexes with molecular targets involved in cell survival, redox regulation, and apoptosis. The secondary aim was to evaluate the direction of interaction among these proteins by integrating analyses of the PTEN/AKT/mTOR/HIF1A survival axis and the KEAP1/YAP/TAZ/CASPASE-3 redox-apoptotic network. The tertiary aim was to determine the toxicity value of MMA and predict the target molecules. The selected proteins were included through a literature-guided, hypothesis-driven strategy because of their reported relevance to residual MMA exposure-related biological responses, particularly oxidative stress, apoptosis, cellular homeostasis, mechanotransduction, and survival-associated tissue signaling.

## 2. Material and Methods

This study aimed to investigate the biocompatibility- and toxicity-related molecular interactions of PMMA, represented by its precursor monomer MMA, with key proteins involved in apoptosis-related signaling and bone osseointegration pathways. A systematic in silico workflow was implemented. MMA–protein interactions were assessed using experimentally resolved three-dimensional protein structures. Subsequently, PPI analysis was performed to contextualize selected targets within an interaction network and infer functional links between pathway components. In parallel in silico toxicity profiling was conducted using specialized predictive tools to estimate potential toxicological risks. Finally, potential MMA-associated target families were investigated using Swiss ADME Target Prediction to complement the structure-based findings. The selected proteins were categorized as follows:

Survival signaling and osteogenic regulation: PTEN, AKT1, and mTOR.

Hypoxia signaling and redox regulation: HIF1A and KEAP1.

Cell death and oxidative stress pathways: GPX4, GPX-PEP1 (GXpep-1-bound structure), and CASP3.

Mechanotransduction and cell adhesion: YAP1, WWTR1 (TAZ), and FERMT2 (Kindlin-2).

The selected proteins were obtained from the Protein Data Bank (PDB) [32], which has a high-resolution three-dimensional structure (ranging from 1.10 Å to 2.50 Å). Subsequently, potential ligand-binding sites were computationally identified to support a consistent docking setup among the targets. Binding pockets and surface voids were characterized using CASTp 3.0 [33], which enabled standardized determination of candidate active sites and void geometries related to ligand docking.

The 11 proteins were selected using literature-based inclusion criteria according to their reported roles in oxidative stress, apoptosis, mechanotransduction, survival signaling, ferroptosis-related regulation, and bone-associated tissue responses.

### 2.1. Preliminary Network Analysis of MMA Toxicity

Protox 3.0 is a web-based virtual toxicity laboratory accessible to academic and noncommercial users for the prediction of multiple toxicological endpoints associated with chemical structures [34]. Protox3.0 incorporates computer-based models trained on real data (in vitro and in vivo) to predict the toxic potential of both existing and virtual compounds [35]. For an input compound, the acute toxicity class and various endpoints are calculated based on chemical similarities to toxic compounds and machine learning models [36]. Protox 3.0 is positioned as a comprehensive and freely accessible computational platform for in silico toxicity prediction, catering to toxicologists, potential regulatory organizations, computational chemists, and medicinal chemists [37]. In this study, the in silico toxicity MMA was investigated using the web-based Protox 3.0 platform (https://tox.charite.de/protox3/index.php?site=home (accessed on 29 April 2026). The results related to the preparation of target proteins were retrieved from the RCSB PDB, and the active sites were determined using CASTp 3.0 (Table 1).

### 2.2. Construction of the PPI Network

The expanded protein–protein interaction (PPI) network was reconstructed de novo in the STRING database (v11.5; https://string-db.org), with the selected proteins serving as seed nodes. Interactions were restricted to Homo sapiens, and a high-confidence interaction score threshold (≥0.700) was applied to ensure reliability. To broaden the interaction landscape beyond the initially selected proteins, first-shell interactors (*n* = 10) were incorporated algorithmically by STRING based on known and predicted protein–protein interactions, while no second-shell interactors were included.

The resulting expanded interactome was exported and analyzed in Cytoscape (v3.10). Network topology was evaluated using the Network Analyzer tool, and the network was treated as an undirected graph. Topological parameters, including degree, betweenness centrality, closeness centrality, clustering coefficient, and network density, were calculated. Proteins were ranked based on degree and betweenness centrality to identify topologically prominent nodes within the expanded network.

In addition, functional enrichment analysis was performed within the STRING platform to evaluate the biological relevance of the network. Specifically, KEGG pathway enrichment analysis was conducted, and enriched pathways were filtered based on false discovery rate (FDR < 0.05) and enrichment strength. Gene Ontology Biological Process (GO-BP) enrichment analysis was also performed to further characterize the functional associations of the network.

The STRING-based expanded PPI network was constructed to identify additional strongly connected and functionally related proteins associated with the manually selected seed proteins within the broader interaction landscape.

### 2.3. Molecular Docking Protocol

Molecular docking simulations were performed using the CB-Dock2 web server. For each PDB structure, the receptor definition was determined individually, and the corresponding chain ID, sequence length, and UniProt accession were recorded. Protein preparation was performed using BIOVIA Discovery Studio by removing crystallographic water molecules and co-crystallized ligands. No additional protonation-state optimization or structural reconstruction was applied.

MMA was retrieved from PubChem and docked using CB-Dock2 with 10 predicted binding cavities per receptor. The binding pose with the lowest predicted Vina score (kcal/mol) was selected for further analysis.

For proteins lacking a well-defined small-molecule binding pocket, docking results were interpreted cautiously as exploration. YAP-related docking was evaluated using the TEAD1–YAP complex (PDB: 4RE1), and TAZ-related docking was evaluated using a TEAD–family structure (PDB: 5HGU), focusing on interface-associated interactions.

### 2.4. Independent Molecular Docking Using AutoDock Vina

To comparatively assess the CB-Dock2 docking results, an independent docking workflow was performed using AutoDock (1.5.6. AutoDock Vina) Vina. For each target, the corresponding experimental 3D structure was retrieved from the PDB, and the same chain selected for Table 3 were retained. All non-protein entities, including crystallographic water molecules, co-crystallized ligands, and other heteroatoms, were removed. No additional protonation-state optimization or structural reconstruction of missing residues or loop regions was performed. Polar hydrogen atoms were added and Kollman united-atom charges were assigned to the receptor using AutoDock Tools, followed by conversion to PDBQT format. The MMA ligand was prepared by energy minimization and exported in PDBQT format prior to docking.

To ensure consistency with the cavity-guided CB-Dock2 protocol, the docking search space in AutoDock Vina was centered on the CB-Dock2-predicted cavity center coordinates (x, y, z) for each receptor. The grid box dimensions were set to encompass the predicted binding cavity (and its surrounding residues) to allow adequate sampling of non-covalent binding poses. AutoDock Vina docking was then executed, and the best-scoring pose (most negative Vina affinity, kcal/mol) was recorded for comparison with CB-Dock2 outputs (Table S1).

### 2.5. Post-Docking Interaction Analysis

The resulting protein–ligand complexes were subjected to detailed interaction mapping. Two-dimensional (2D) and three-dimensional (3D) visualizations were generated using BIOVIA Discovery Studio Visualizer to annotate non-covalent interactions, including conventional hydrogen bonds, carbon–hydrogen bonds, and hydrophobic contacts.

### 2.6. Swiss ADME and Swiss Target Prediction Analysis

To complement the structure-based docking results and explore the broader interactome of MMA, ligand-based target prediction was performed using the Swiss ADME (version 1.0, 2023) and SWİSS Target Prediction software (version 2.0, 2019) program. The chemical structure of MMA (CID: 6658) was queried against the Homo sapiens database. SWISS ADME was used because it generates descriptive-based mathematical models that allow the prediction of toxicological properties from compound structures. By analyzing molecular properties with SWISS Target, it identifies potential interaction groups, thus providing useful information about possible toxicological outcomes. Including toxicity assessment as an integral component of the study allows for a broader evaluation of the potential risks associated with MMA and PMMA in dental applications, supporting more informed material selection and patient safety considerations.

## 3. Results

### 3.1. Selection and Structural Curation of Target Proteins

To elucidate the molecular mechanisms potentially underlying MMA-associated biocompatibility, a systematic panel of 11 key proteins was curated, representing biologically relevant nodes involved in oxidative stress, apoptosis, cellular homeostasis, mechanotransduction, and related tissue response signaling pathways.

The structural characteristics and biological rationale for the selection of each target are detailed in Table 1. Computational characterization of the putative ligand-binding domains and active site residues was conducted using CASTp 3.0, facilitating a standardized delineation of the pocket geometries required for ligand accommodation. 

### 3.2. In Silico Toxicity Profiling of MMA

**Figure 1:**
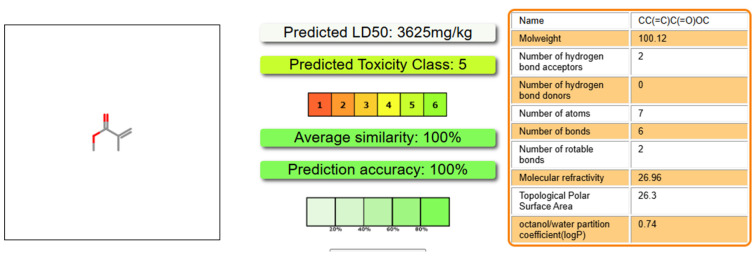
Predicted toxicity of MMA.

The systemic toxicity profile of MMA was evaluated using the ProTox-3.0 platform to identify potential physiological risks. Predictive analysis estimated an LD50 value of 3625 mg/kg, indicating that it falls within the low-toxicity category in its self-numbered classification. (Figure 1).

The key toxicological findings, summarized in Table 2, were interpreted in the context of the probability-based nature of ProTox-3.0 predictions. MMA showed a predicted active signal for respiratory toxicity (probability: 0.70) and brain–blood barrier (BBB) permeability (probability: 0.90). A predicted active signal was also observed for CYP2C9 interaction (probability: 0.64); however, this result was interpreted cautiously because of its moderate probability. In contrast, cardiotoxicity, hepatotoxicity, nephrotoxicity, and neurotoxicity were predicted as inactive, although some of these endpoints showed relatively low probability values, indicating limited predictive confidence.

Overall, the ProTox-3.0 results probability values indicate the reliability of the model, and the active values are listed in the table. These findings should be considered as in silico indicators rather than definitive evidence of organ-specific toxicity or metabolic liability, and their biological and toxicological relevance requires future experimental confirmation.

### 3.3. PPI Network Topology and Hub Protein Identification

**Figure 2:**
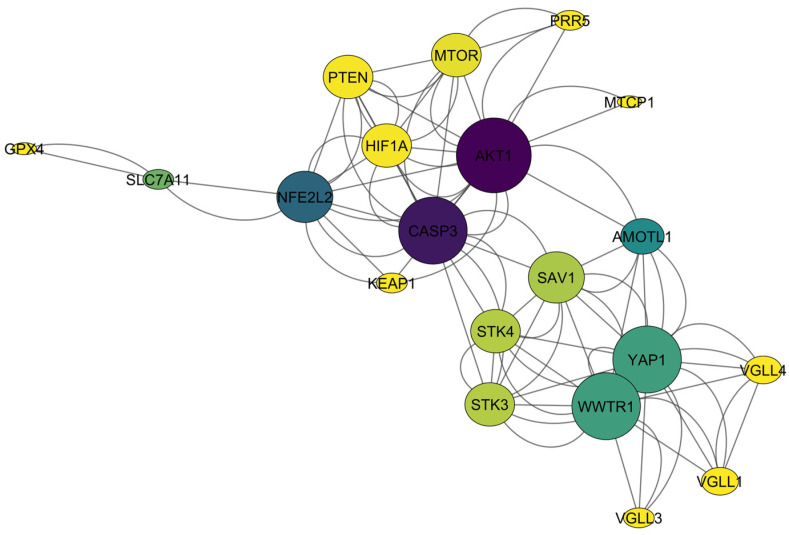
Extended protein–protein interaction network created using the STRING database and visualized in Cytoscape. Nodes represent proteins, and edges represent functional and physical interactions. Node size reflects degree centrality, while node color reflects intercalation centrality, highlighting topologically distinct nodes within the extended interaction network.

The PPI network, constructed via STRING and analyzed with Cytoscape, identified 20 key targets of unknown biological response associated with the 11 selected proteins. These targets play a crucial role in the molecular dialog that may influence osseointegration and cell viability. The topological parameters of the network (Table 3) highlight potential regulators:

In the extended protein–protein interaction network, AKT1 (Figure 2) is the most connected node, with a degree of 18 and the highest central proximity value of 0.575. Following in terms of node number values are CASP3, WWTR1, and YAP1, each with a degree of 16, while SAV1 and NFE2L2 (both with a degree of 12) are identified as important interconnected components of the extended interaction network. mTOR, PTEN, and HIF1A follow with a value of 10. A more detailed assessment of node importance using mediator centrality revealed that AKT1 ranked highest with a value of (0.327), followed by CASP3 (0.301), NFE2L2 (0.215), AMOTL1 (0.170), and WWTR1 (0.141). These findings demonstrate that AKT1 and CASP3 are not only highly linked nodes but also key bridging elements that can facilitate communication between different subnetworks.

In the extended network analysis, WWTR1, YAP1, SAV1, STK3, and STK4 were associated with mechanotransduction, while NFE2L2, KEAP1, GPX4, and GXpep-1 stood out among the nodes associated with oxidative stress regulation and ferroptosis.

**Figure 3:**
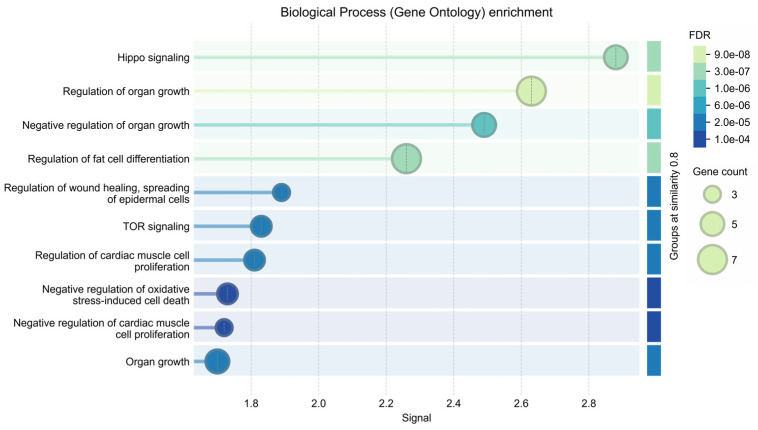
Gene Ontology Biological Process enrichment analysis of the expanded interactome. The lollipop plot illustrates the most significantly enriched biological processes. The x-axis represents enrichment magnitude, circle size corresponds to gene count, and color indicates the false discovery rate (FDR).

Complementary Gene Ontology Biological Process enrichment analysis (Figure 3) showed that HIPPO signaling pathway-associated YAP/TAZ protein companions have the strongest response, followed by the cell survival proteins AKT and negative regulatory caspase groups, which also gave strong signals. These findings are consistent with protein–protein interaction data.

**Figure 4:**
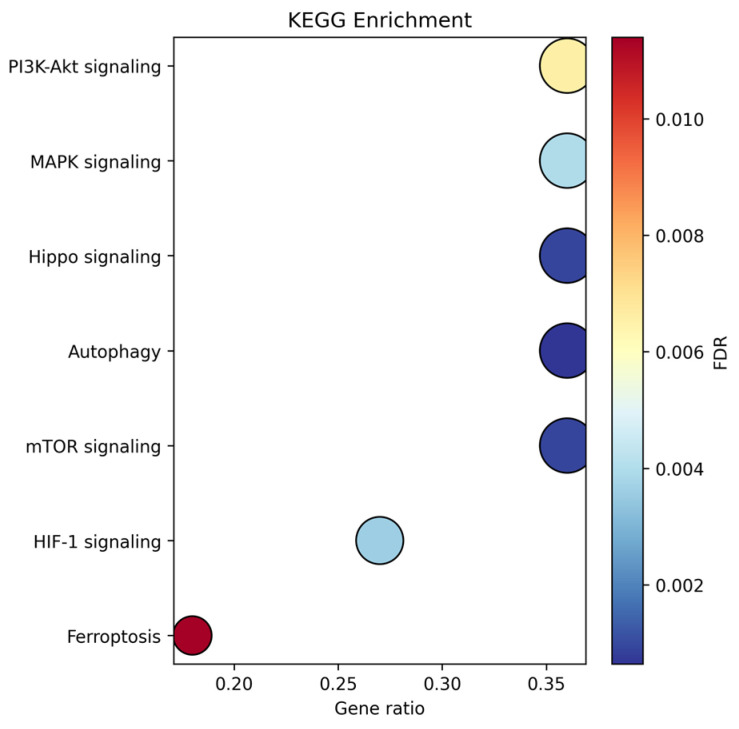
KEGG pathway enrichment analysis of the selected protein set. KEGG pathway enrichment analysis of the expanded interactome. The dot plot illustrates significantly enriched KEGG pathways identified using the STRING database. The x-axis represents gene ratio, the y-axis lists enriched pathways, dot size corresponds to gene count, and color indicates the false discovery rate (FDR). Pathways with FDR < 0.05 were considered statistically significant.

To validate the biological significance of the expanded network and minimize potential selection bias, functional enrichment analyses were performed. KEGG pathway enrichment analysis (Figure 4) revealed that the PI3K–AKT, MAPK, Hippo, mTOR, and HIF1A signaling pathways, which are related to cell survival, stress response, and cellular homeostasis, showed the most statistically significant associations (FDR < 0.05).

This is the pathway strongly associated with this protein set, as it has both the highest “gene ratio” value (far right) and the lowest FDR value (red). The HIF-1 signaling pathway stands out with its large dot size (high gene number), while the mTOR, autophagy, and Hippo signaling pathways were also statistically significantly enriched.

### 3.4. Molecular Docking and Pathway Interconnectivity

Molecular docking simulations showed that YAP, TAZ, and mTOR proteins gave a moderate (−4.2) binding signal. This binding score was most closely followed by PTEN, AKT and GPX, with a score of −4.0 (Table 4). Molecular docking data were supported by protein–protein interaction and KEGG data. For comparative reference, AutoDock Vina estimates for MMA against each analyzed protein target were additionally calculated and are provided in Supplementary Table S1.

**Figure 5:**
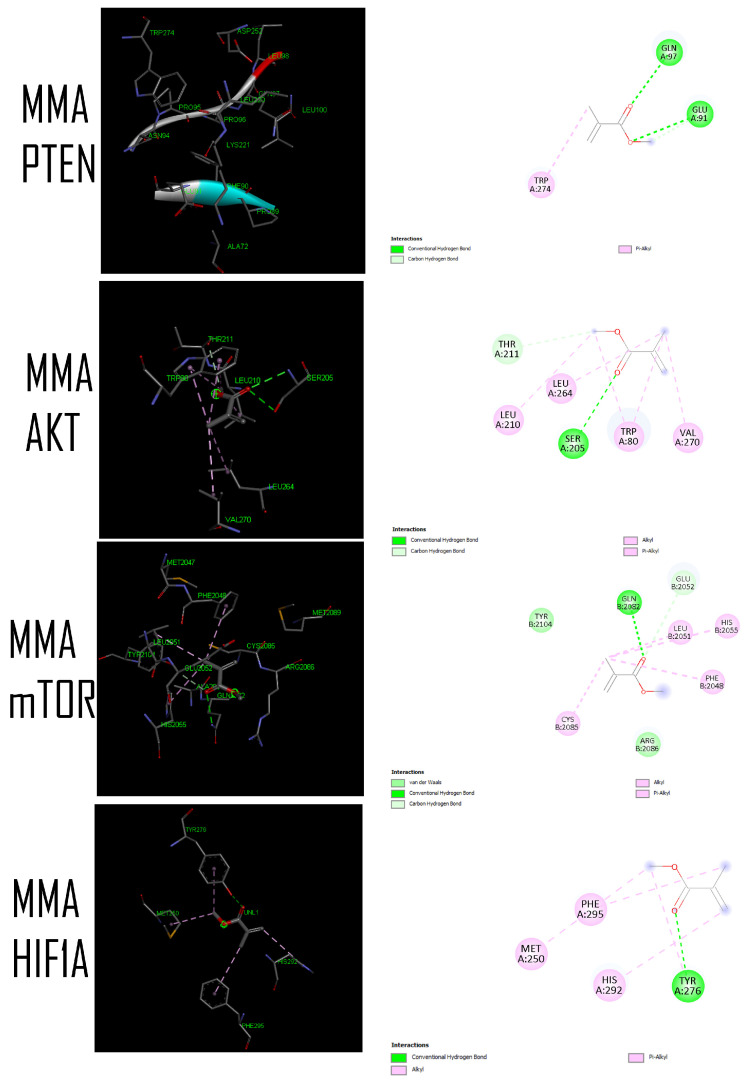
Representative 2D and 3D interaction maps of MMA with selected survival signaling-related proteins. The (**left**) panels show the 3D view of the protein–MMA complex, and the (**right**) panels show the corresponding 2D interaction diagrams.

**Figure 6:**
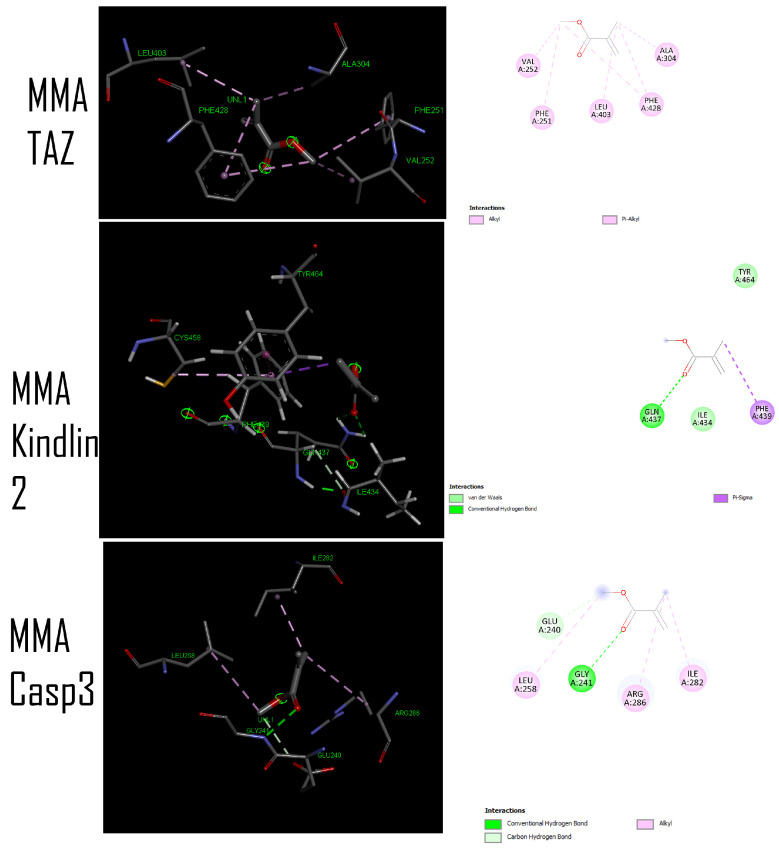
Representative 2D and 3D interaction maps of MMA with selected apoptosis-related and adhesion-related proteins. The (**left**) panels show the 3D view of the protein–MMA complex, and the (**right**) panels show the corresponding 2D interaction diagrams.

**Figure 7:**
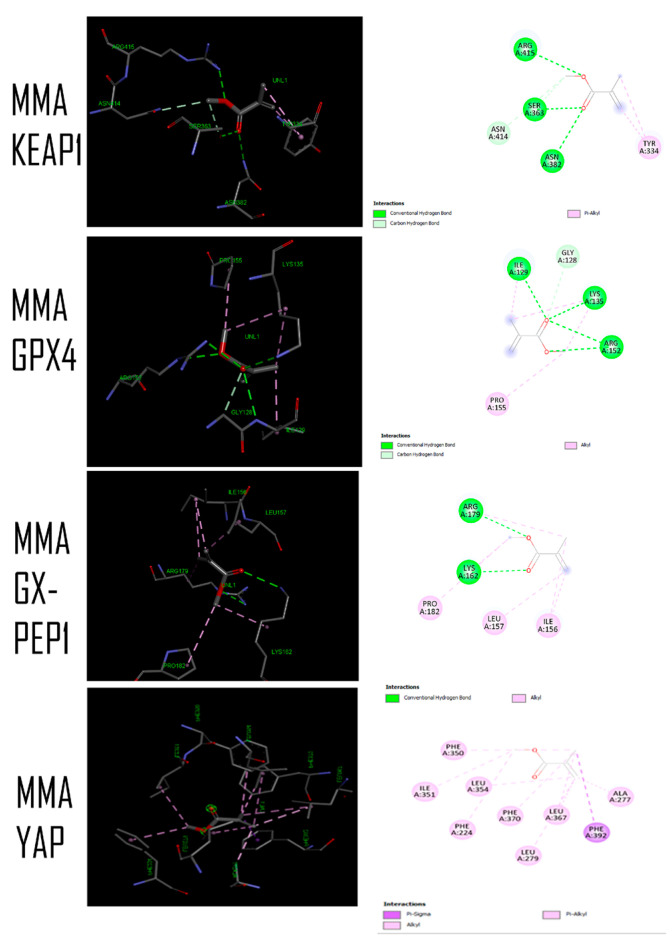
Representative 2D and 3D interaction maps of MMA with selected oxidative stress-related and mechanotransduction-related proteins. The (**left**) panels show the 3D view of the protein–MMA complex, and the (**right**) panels show the corresponding 2D interaction diagrams.

Representative 2D/3D interaction diagrams for the top-ranked poses are provided in Figure 5, Figure 6 and Figure 7.

### 3.5. Pharmacophore Modeling and Residue-Specific Interaction Analysis

Residue-level interaction analysis was performed to characterize the non-covalent interaction patterns between MMA and selected protein targets. The 2D and 3D interaction maps (Figure 5, Figure 6 and Figure 7) demonstrate that MMA is primarily stabilized through a combination of hydrogen bonding and hydrophobic interactions across different protein environments.

Molecular docking of MMA with PTEN protein showed conventional hydrogen bonding with amino acid residues GLN A:97 and GLU A:91, while bonding appears to occur with the pi-alkali residue TRP A:274. AKT1 showed a strong conventional hydrogen bond with the amino acid residue SER A:205 and a weak hydrogen bond with THR A:211, while weak bonding appears to occur with the pi-alkali residues: LEU A:210-264, TRP A:80, and VAL A:270. Among the analyzed targets, mTOR showed a strong conventional hydrogen bond with residue GLN B:2082, weak hydrogen bonds with residues TYR B:2104 and GLU B:2052, and weak pi-alkali bonding.

It also showed hydrophobic contacts involving residues such as LEU B:2051, HIS B:2055, PHE B:2048, and CYS B:2085. Similarly, HIF1A was observed to have strong hydrogen bonding with TYR A:276, while it had weak pi-alkali bonding with HIS A:292, PHE A:295, and MET A:250 (Figure 5).

In the TAZ complex, MMA showed weak bonding with alkyl and π-related contacts involving residues such as PHE, LEU, VAL, and ALA. In the Kindlin2 protein, it formed hydrogen bonds with amino acid residues GLN A:437, ILE A:434, and TYR A:464, as well as a strong covalent bond with PHE A:439 via pi-sigma. The Caspase–MMA molecular coupling was established via hydrogen bonds with residues GLY A:241 and GLU A:240, and via alkaline bonds with residues LEU, ARG, and ILE (Figure 6).

In the KEAP1 complex, MMA formed conventional hydrogen bonds with ARG A:415, SER A:363, and ASN A:382, while a carbon-mediated hydrogen bond was formed with residue ASN A:414. A pi-alkali bond was present with TYR A:434.

In the GPX4 complex, ILE A:129, LYS A:135, and ARG A:152 form conventional hydrogen bonds, while a carbon-mediated hydrogen bond is formed with GLY A:128. An alkali bond is present with PRO A:155. In the GXPEP1 complex, conventional hydrogen bonds are observed with residues ARG A:179 and LYS A:172, while alkali bonds are observed with residues PRO, ILE, and LEU. In the YAP complex, no hydrogen bonds are formed; however, alkali and pi-alkali bonds are formed with amino acid residues PHE, LEU, and ALA (Figure 7).

### 3.6. Putative Target Identification via Swiss ADME Prediction

SwissADME analysis showed that MMA has a low molecular weight (100.12 g/mol) and moderate lipophilicity, with a cLogP (XLOGP3) value of 1.38 and a cLogS (SILICOS-IT) value of −0.71, indicating acceptable aqueous solubility. The compound exhibited a topological polar surface area (TPSA) of 26.30 Å^2^, two hydrogen bond acceptors, zero hydrogen bond donors, and two rotatable bonds, consistent with a small and structurally simple molecule. In the pharmacokinetic assessment, MMA showed high gastrointestinal absorption, predicted blood–brain barrier permeability, and a bioavailability score of 0.55. Drug-likeness evaluation indicated compliance with the Lipinski, Veber, and Egan criteria, whereas Ghose and Muegge filters were not fully satisfied, mainly due to its low molecular weight and limited molecular complexity. In the medicinal chemistry assessment, MMA showed zero PAINS alerts, one Brenk alert (michael_acceptor_1), and a synthetic accessibility score of 1.12, suggesting a chemically simple and readily accessible structure. (Table 5). In the SWISS TARGET target analysis, MMA was not shown to interact with the target molecules; therefore, these results are presented as additional data (Table S2).

## 4. Discussion

In a recent in silico study by Saini et al. [2], PMMA-based materials were found to exhibit strong binding affinities to key receptors involved in bone metabolism, including RANKL (nuclear factor-kappa B ligand receptor activator), bone morphogenetic proteins (BMPs), fibronectin, and osteoprotegerin. In particular, the observed interactions between PMMA and alkaline phosphatase (ALP) and tartrate-resistant acid phosphatase (TRAP) receptors suggest that PMMA may play a role in cellular resonses to oxidative stress and in the osteointegration process. These findings demonstrate that PMMA can contribute to the osteointegration process through interactions with osteogenic signaling pathways at the molecular level, beyond providing structural stability in dental restorations [2]. Inspired by this work, proteins involved in the PTEN/mTOR pathway, which plays a role in cell viability and metabolism, YAP/TAZ proteins, which are among the novel targets in oxidative stress regulation, and ferroptotic apoptotic process proteins involved in cellular repair mechanisms were included among our targets. Previous studies have shown that MMA exposure can increase reactive oxygen species (ROS) levels and decrease intracellular glutathione (GSH), thus contributing to oxidative stress [38,39]. Oxidative imbalance has also been associated with GPX4 inactivation, lipid peroxide accumulation, osteoblast death, and ferroptosis-related bone loss [40,41,42,43,44]. Consistent with this framework, the prominence of YAP1, WWTR1 (TAZ), NFE2L2, and GPX4 among the central and interacting proteins in the PPI analysis may suggest the relevance of oxidative stress-regulatory and ferroptosis-associated signaling contexts within the expanded network, although this should not be interpreted as evidence of direct MMA-specific pathway modulation.

Disruption of cellular homeostasis can activate p53/PTEN-related signaling, suppress the AKT/mTOR pathway, inhibit proliferation, and promote apoptosis in osteoblast-related contexts [25,27,45,46]. Furthermore, it has been reported that PMMA-induced oxidative DNA damage can suppress AKT/mTOR signaling via PTEN activation under conditions of methacrylate monomer exposure [47]. In this study, molecular docking suggested weak but detectable interaction trends between MMA and proteins such as mTOR, AKT1, and PTEN, whereas KEGG enrichment and PPI analyses placed these proteins within a broader context of biologically related signaling. Similarly, the prominence of CASP3 in the PPI network is consistent with previous reports showing that MMA-induced oxidative stress can trigger p53/PTEN-mediated apoptosis via CASP3 activation [38,48].

YAP/TAZ are well-known regulators of oxidative stress, tissue microenvironment sensing, and osteogenic differentiation [31,49,50,51]. In our molecular docking and pharmacophore analysis, YAP/TAZ-related structural templates showed weak but relatively favorable interaction tendencies, including alkyl/pi-alkyl contacts. However, these findings should be interpreted only as exploratory structural observations and not as evidence of direct modulation of mechano-responsive signaling proteins by MMA. Interactions involving residues such as VAL A:252 and LEU A:403 in the TAZ-related template can therefore be considered as weak exploratory signals in a mechanotransduction-related context rather than direct evidence of functional modulation [52]. The formation of van der Waals and hydrophobic contacts between MMA and TYR A:113 in the mTOR-related structure may indicate a possible structural interaction trend related to signaling concerning cell metabolism and protein synthesis; however, this should not be considered a proven biological effect [53,54,55,56].

More generally, pharmacophore and docking analyses have shown that MMA interacts with multiple targets via relatively weak but repetitive non-covalent contacts, primarily involving hydrogen bonds and hydrophobic interactions. The carbonyl group contributes predominantly as a hydrogen bond acceptor, while the methyl group is associated with hydrophobic contacts in receptor environments. These patterns may indicate a tendency toward interaction at the multiple target and pathway level rather than strong receptor-specific binding. Given the modest docking affinities observed, these results should best be interpreted as exploratory and hypothetical.

MMA is a respiratory irritant and skin sensitizer associated with occupational asthma in a small number of case reports. Numerous in silico and in chemico studies suggest that MMA is unlikely to be a respiratory sensitizer, whereas a small number of in vitro studies suggest that MMA generally has weak effects [57]. The in silico toxicity assessment of MMA using ProTox-3.0 showed a low acute toxicity profile (LD50: 3625 mg/kg; Toxicity Class 5), which is largely consistent with previous toxicological reports under controlled exposure conditions. The estimated respiratory toxicity is consistent with documented evidence of mucosal irritation and occupational respiratory effects associated with MMA vapor inhalation [58].

No hepatotoxic, nephrotoxic, cardiotoxic, mutagenic, or carcinogenic signals were detected in the Protox toxicity findings; this is consistent with previous genotoxicity assessments showing minimal long-term systemic risk [58].

A study in which PMMA-based resin monomers were examined using computational methods reported that neurotoxicity levels could reach 90% [59]. The presence of blood–brain barrier permeability in the Swiss ADME and target targets suggests that PMMA exposure may have adverse effects on brain tissue in experimental models. Previous studies have reported that PMMA can leak various toxic compounds, and these compounds can pass through biological membranes, causing adverse effects locally or beyond the oral cavity [60]. The high GI absorption in the SWISS ADME results supports the idea that it can penetrate other tissues by affecting systemic spread through oral absorption.

The CYP2C9 enzyme group is an important member of the p450 cytochrome family and is metabolized through oxidative stress and hydrolysis [61]. The activity of this enzyme in our study supports the idea that the oxidative stress pathway is active and may affect the toxicity profile. Although MMA is known as a respiratory irritant and dermal sensitizer, the overall weight of current in silico, in vitro, epidemiological and occupational medicine data indicates that it is not a strong respiratory sensitizer. Reported respiratory complaints appear to be mostly related to irritation due to short-term high exposure, whereas case reports of asthma or respiratory sensitization are limited, involve mixed exposure and have low evidential power [57]. The data of our study show that respiratory interactions are active.

These findings should be interpreted within a seed-protein-centered analytical framework. The expanded STRING network was used to identify additional topologically prominent and functionally related proteins associated with the selected targets rather than to infer an unbiased MMA-specific interactome. Accordingly, the observed enrichment of pathways such as PI3K-Akt, Hippo, apoptosis, and oxidative stress may partly reflect the biological characteristics of the initial seed set; however, the emergence of additional prominent nodes still provides a broader contextual basis for prioritizing pathway-relevant proteins for future mechanistic investigation.

### Limitations and Clinical Implications

Molecular docking is widely used to predict ligand-binding modes; however, its scoring functions have well-documented limitations in accurately estimating binding affinities. Previous large-scale evaluations have demonstrated that docking scores often fail to reliably rank compounds according to their experimental affinities, and no single docking program performs consistently across different targets [62]. Moreover, despite continuous methodological improvements, the development of universally accurate docking and scoring approaches remains an ongoing challenge [63]. Importantly, the top-ranked docking pose does not always correspond to the experimentally correct binding mode, particularly in cross-docking scenarios. Therefore, selection of the best solution should not rely solely on scoring energy but should also consider structural context [64]. In this regard, the docking results obtained in the present study should be interpreted as indicative of possible ligand–protein interactions rather than definitive measures of binding affinity.

In addition, the potential covalent reactivity of MMA, particularly via Michael-type addition to nucleophilic residues such as cysteines, was not modeled in the present study. Therefore, the docking results reflect only non-covalent interaction tendencies and should be interpreted as complementary to, rather than representative of, the full spectrum of MMA–protein interactions [64].

From a clinical perspective, these findings should be regarded as exploratory in silico observations rather than evidence of direct clinical effects of MMA exposure. Nevertheless, they may help identify biologically relevant pathways related to residual monomer release, oxidative stress, and local tissue response, thereby guiding future material optimization and targeted experimental validation.

## 5. Conclusions

This study presents a comprehensive in silico and bioinformatics-based evaluation of the potential molecular relationships of MMA, the main monomer of PMMA-based dental materials, with 11 selected proteins involved in signaling pathways that indirectly affect bone integration, including oxidative stress regulation, apoptosis, hypoxia response, and mechanotransduction. To the best of our knowledge, this is the first study to examine MMA within such an integrated framework by combining molecular docking, expanded PPI analysis, topological network assessment, KEGG/GO enrichment, toxicity prediction, and ligand-based target profiling. In this context, the study was conducted to evaluate the biocompatible and safe form of MMA in terms of how it can bind to biologically relevant signaling networks at the substance–tissue interface in a pre-laboratory setting.

The findings indicate that MMA should not be regarded solely as a passive structural component but rather as a chemically active monomer that may be associated with multiple interconnected regulatory pathways. In particular, the expanded network analysis highlighted topologically prominent nodes such as AKT1, CASP3, NFE2L2, YAP1, and WWTR1, while docking analysis showed comparatively interaction tendencies for mTOR- and YAP/TAZ-related structures within the analyzed protein panel. The analysis revealed distinct molecular behaviors of MMA, elucidating their stable structures and promising biocompatibility. Furthermore, pharmacophore modeling identified key functional groups involved in critical interactions, such as carbonyl, hydroxyl, and methyl groups, offering a deeper understanding of their role in biocompatibility. These findings highlight the potential of computational approaches in guiding the design and optimization of dental prosthetic materials with enhanced biocompatibility. However, while our study offers valuable insights, further experimental validation is necessary to confirm the accuracy of these outcomes.

Clinicians and technicians involved in dental prosthetics can leverage the insights provided by this research to make informed decisions regarding the selection and utilization of PMMA-based materials. By considering factors such as structural stability, molecular interactions, and safety considerations, practitioners can contribute to the advancement of biocompatible dental materials, ultimately improving patient outcomes and satisfaction.

## Figures and Tables

**Table 1:** Results related to the preparation of target proteins retrieved from the RCSB PDB, with the active sites determined using CASTp 3.0.

Name	PDB/UniProt ID	Resolution (Å)	Chain	Weight (kDa)	Sequence Length	Active Site (Residue Number)	Biological Relevance of Each Target
PTEN	1D5R	2.10	A	38.65	324	16, 24, 92, 93, 124, 125, 126, 127, 128, 129, 130, 159 160, 162, 164, 167, 168, 171	Central negative potential regulator of AKT signaling; recently implicated in osteoimmune modulation and peri-implant bone formation.
AKT1	4EJN	2.19	A	52.45	446	13, 14, 15, 16, 17, 18, 20, 21, 22, 25, 27, 28, 30, 32, 34, 36, 37, 38, 50, 51, 52, 53, 54, 55, 56, 58, 59, 60, 68, 69, 72, 77, 79, 80, 81, 82, 83, 84, 86, 87, 88, 89, 156, 157, 158, 159, 161, 164, 181, 203, 204, 205, 207, 210, 211, 212, 229, 234, 261, 264, 268, 270, 271, 272, 273, 274, 275, 276, 277, 278, 279, 281, 290, 291 ,292, 293, 294, 295, 296, 297, 298, 299, 308, 309, 310, 311, 312, 31, 316, 317, 320, 323, 324, 325, 326, 327, 330, 331, 333, 334	Key survival kinase linking metabolic adaptation, osteoblast viability, and angiogenic signaling.
mTOR	4DRI	1.45	B	28.36	98	52, 57, 67, 68, 6970, 72, 73, 75, 77, 78, 79, 80, 84, 85, 86, 87, 88, 90, 91 112, 113, 116, 117, 118, 119, 120, 121, 122, 130	Co-crystal structure of the PPIase domain of FKBP51, rapamycin and the FRB fragment of mTOR. Master regulator of cellular growth and osteogenic metabolism.
HIF1A	4H6J	1.52	A	26.69	113	324, 325, 326, 327, 328, 333, 334	Integrates hypoxia-driven angiogenesis with osteogenic adaptation.
KEAP1	4L7B	2.41	A	66.78	300	334, 336, 337, 363, 364, 365, 366, 367, 368, 369, 380, 382, 383, 384, 385, 386, 387, 389, 414, 415, 416, 417, 418, 419, 420, 431, 433, 434, 435, 436, 450, 460, 461, 462, 463, 464, 465, 466, 467, 478, 479, 480	Redox sensor protein regulating oxidative stress-dependent cell survival.
GPX4	2OBI	1.55	A	20.96	183	127, 128, 129, 135, 139, 152, 153, 154, 155, 157	Key inhibitor of ferroptosis; recently linked to implant-induced oxidative stress responses.
GX-PEP1 (GXpep-1-bound structure)	5H5Q	1.10	A	21.34	169	152, 154, 155, 156, 157, 158, 162, 166, 179, 180, 181, 182, 183, 184	Included as an alternative ligand-bound structural state of GPX4 to examine whether MMA docking behavior is consistent across different conformational templates.
TEAD1–YAP complex	4RE1	2.20	A	83.01	220	194, 195, 197, 198, 199, 200, 202, 237, 238, 239, 266, 267, 268, 269, 270, 271, 383, 384, 385, 407, 408	Key downstream effector of Hippo signaling and mechanotransduction-related transcriptional regulation.
TEAD2-palmitate TAZ	5HGU	2.05	A	54.77	237	281, 283, 288, 291, 292, 326, 327, 340, 342, 344, 346, 353 356, 357, 358, 359, 360, 361, 362, 374, 375, 376, 377, 378, 379, 380, 381, 384, 411, 413, 415, 418, 420	TAZ (WWTR1): Key mediator of Hippo signaling and mechanotransduction, with relevance to osteogenic differentiation and biomaterial-responsive cellular signaling.
Kindlin-2 (FERMT2)	2LKO	2.10	A	55.47	474	102, 104, 106, 141, 143, 144, 236, 238, 240, 242, 245, 246, 248, 249, 252, 271, 273, 274, 275, 277, 297, 300, 301, 304, 305, 306, 307, 308, 562, 567, 568, 569, 570 571, 572, 600, 601, 649	Essential for integrin-mediated osteoblast adhesion to implant surfaces.
Caspase-3	1PAU	2.50	A	29.04	147	294, 295	Final executioner of apoptosis; key indicator of PMMA/MMA-induced cytotoxic signaling.

YAP interface context (PDB: 4RE1), not as an isolated YAP receptor.

**Table 2:** Key toxicological outcomes predicted by ProTox-3.0 for MMA.

Classification	Target	Shorthand	Prediction	Probability
			MMA	MMA
**Metabolism**	Cytochrome CYP2C9	CYP2C9	Active	0.64
**Organ toxicity**	Respiratory toxicity	respi	Active	0.70
**Toxicity endpoints**	BBB-barrier	bbb	Active	0.90

**Table 3:** Topologically prominent nodes identified in the expanded STRING-derived PPI network.

Gene	Degree	Closeness Centrality	Betweenness Centrality	Neighborhood Connectivity
AKT1	18	0.575	0.327	4.22
CASP3	16	0.558	0.301	5.75
WWTR1	16	0.452	0.141	4.5
YAP1	16	0.452	0.141	4.5
SAV1	12	0.5	0.060	6.33
NFE2L2	12	0.475	0.215	5.16
STK3	10	0.487	0.053	7.0
STK4	10	0.487	0.053	7.0
mTOR	10	0.431	0.016	5.8
PTEN	10	0.452	0.004	6.6
HIF1A	10	0.452	0.004	6.6
AMOTL1	8	0.513	0.170	7.75
CASP3	8	0.76	0.13	6.50
AKT1	7	0.68	0.00	6.25
SAV1	6	1.00	0.11	6.00
VGLL1	6	0.327	0.0	6.33
VGLL4	6	0.327	0.0	6.33
PTEN	5	1.00	0.00	5.80
HIF1A	5	0.80	0.00	5.80
WWTR1	5	1.00	0.00	5.75
YAP1	5	0.80	0.00	5.37
STK3	5	1.00	0.03	5.33
STK4	5	1.00	0.03	5.00
SLC7A11	4	0.339	0.105	3.5
mTOR	4	1.00	0.00	5.00
KEAP1	4	0.395	0.0	7.5
PRR5	4	0.379	0.0	7.0
VGLL3	4	0.322	0.0	8.0
GX-PEP1 (GXpep-1-bound structure)	2	0.40	0.00	3.50
GPX4	1	1.00	0.00	2.00

**Table 4:** Molecular docking results of the core target proteins with MMA.

Target (Gene/Protein)	PDB ID	Chain	CB-Dock Cavity Rank	Best Pose (Vina Mode)	Binding Affinity (kcal/mol)	RMSD l.b. (Å)	RMSD u.b. (Å)	Best Cavity ID	Cavity Size (x, y, z)	Cavity Center (x, y, z)
PTEN	1d5r	A	1	1	−4.00	0.00	0.00	1	16, 16, 16	44.771 78.910 29.029
AKT1	4ejn	A	1	1	−4.10	0.00	0.00	1	15, 15, 15	29.817 43.243 15.125
mTOR	4dri	B	1	1	−4.20	0.00	0.00	2	16, 16, 16	35 43 36
HIF1A	4h6j	A	1	1	−3.00	0.00	0.00	1	16, 16, 16	19.087 −17.259 −34.909
KEAP1	4l7b	A	1	1	−3.90	0.00	0.00	1	22, 23, 32	7.472 −14.479 −14.411
GX-PEP1 (GXpep-1-bound structure)	5h5q	A	1	1	−4.00	0.00	0.00	3	16, 16, 16	7.203 11.455 −6.512
GPX4	2obi	A	1	1	−3.90	0.00	0.00	1	16, 16, 16	32.320 −27.856 −8.825
YAP	4re1	A	1	1	−4.20	0.00	0.00	1	16, 23, 16	17.210 −6.130 −20.962
TAZ	5hgu	A	1	1	−4.20	0.00	0.00	1	16, 16, 16	−9.622 15.434 92.162
Kindlin-2	2lko	A	1	1	−3.8	0.00	0.00	7	16, 16, 16	−15 −7 −4
CASP3	1pau	A	1	1	−3.50	0.00	0.00	1	16, 16, 16	32.458 96.173 8.113

Quantitative docking parameters, including cavity rank, best pose, binding affinity, and cavity geometry, are summarized in Table 4.

**Table 5:** Swiss ADME analysis results for MMA.

Parameter	MMA
Formula	C_5_H_8_O_2_
Molecular weight (g/mol)	100.12
cLogP (XLOGP3)	1.38
cLogS (SILICOS-IT)	−0.71
TPSA (Å^2^)	26.30
H-bond acceptors	2
H-bond donors	0
Rotatable bonds	2
Heavy atoms	7
Fraction Csp3	0.40
Molar refractivity	26.96
GI absorption	High
BBB permeant	Yes
Bioavailability score	0.55
Lipinski	Yes; 0 violation
Ghose	No; 3 violations: MW < 160, MR < 40, #atoms < 20
Veber	Yes
Egan	Yes
Muegge	No; 1 violation: MW < 200
PAINS	0 alert
Brenk	1 alert: michael_acceptor_1
Leadlikeness	No; 1 violation: MW < 250
Synthetic accessibility	1.12

Graph gastrointestinal, BBB: blood–brain barrier.

## Data Availability

Data are available from the corresponding author upon reasonable request.

## Supplementary Materials

The following supporting information can be downloaded at: https://www.mdpi.com/article/10.3390/cimb48060630/s1.

## Abbreviations

MMA: methyl methacrylate, PMMA: polymethyl methacrylate, CB-Dock: accurate protein–ligand blind docking tool, mTORC1: rapamycin and the FRB fragment of mTOR, PTEN: phosphatase and tensin homolog, Akt: protein kinase B, CasP3: caspase 3 protein, YAP: Yes-associated protein 1, TAZ: TEAD–family-associated structural template GPX4: glutathione peroxidase 4, GX-PEP1 (GXpep-1-bound structure): GPX4 ligand-bound structural template, HIF1a: hypoxia-inducible factor 1-alpha, KEAP1: Kelch-like ECH-associated protein 1, Kindlin-2 (FERMT2): fermitin family homolog 2, PMMA: polymethyl methacrylate, ROS: reactive oxygen species, PPI: protein–protein interaction, EGFR: epidermal growth factor receptor, CA: carbonic anhydrase, BMPs: bone morphogenetic proteins, ALP: alkaline phosphatase, TRAP: tartrate-resistant acid phosphatase, GSH: glutathione, NRF2: nuclear factor erythroid 2-related factor 2, PDB: Protein Data Bank, FDR: false discovery rate, ECM: extracellular matrix, VAL: valine, PHE: phenylalanine, LEU: leucine, ALA: alanine. TRY: tyrosine, SER: serine, ARG: arginine, GI: gastrointestinal, and BBB: blood–brain barrier.
