# Supplementary material for: Bioactive Assessment of MMA-Based Dental Materials: Molecular Docking and Network Topology Analysis of Stress-Regulated Survival, Apoptosis, and Mechanotransduction Pathways
Source: Curr Issues Mol Biol. 2026 Jun 17;48(6):630. doi: 10.3390/cimb48060630 (PMC13297651; doi:10.3390/cimb48060630)
Supplement: Supplementary file 1 [file cimb-48-00630-s001.zip › cimb-4321633-supplementary.pdf]

Table S1. AutoDock Vina docking scores for MMA against selected targets.

Target	PDB ID	Grid center (x,y,z)	Box size (x,y,z)	Exhaustiveness	Best affinity(kcal/mol)	RMSD (lb/ub)
PTEN	1d5r	44.771<br>78.910<br>29.029	40,40,40	8	-3.9	0.000
AKT1	4ejn	29.817<br>43.243<br>15.125	35,35,35	8	-4.0	0.000
mTOR	4dri	35<br>43<br>36	35,35,35	8	-4.4	0.000
HIF1A	4h6j	19.087<br>-17.259<br>-34.909	35,35,35	8	-3.1	0.000
KEAP1	4l7b	7.472<br>-14.479<br>-14.411	35,35,35	8	-3.9	0.000
GX-PEP1 (GXpep-1-bound structure)	5h5q	7.203<br>11.455<br>-6.512	40,40,40	8	-3.6	0.000
GPX4	2obi	32.320<br>-27.856<br>-8.825	35,35,35	8	-3.6	0.000
YAP	4re1	17.210<br>-6.130<br>-20.962	35,35,35	8	-4.1	0.000
TAZ	5hgu	-9.622<br>15.434<br>92.162	35,35,35	8	-4.2	0.000
Kindlin-2	2lko	-15<br>-7<br>-4	35,35,35	8	-3.9	0.000
CASP3	1pau	32.458<br>96.173<br>8.113	40,40,40	8	-3.4	0.000

Table S2. SwissTargetPrediction results for MMA, including target classes and reported probability values.

**Target**	**Common Name**	**Uniprot ID**	**Target Class**	**Probability***	**Known Actives (3D/2D)**	**Gene Symbol**	**Is Study Target**
Hydroxycarboxylic acid receptor 2	HCAR2	Q8TDS4	Family A G protein-coupled receptor	0	0 / 5	HCAR2	No

Transient receptor potential cation channel subfamily A member 1	TRPA1	O75762	Voltage-gated ion channel	0	1 / 0	TRPA1	No
Carbonic anhydrase II	CA2	P00918	Lyase	0	1 / 0	CA2	No
Carbonic anhydrase VII	CA7	P43166	Lyase	0	1 / 0	CA7	No
Carbonic anhydrase XII	CA12	O43570	Lyase	0	1 / 0	CA12	No
Carbonic anhydrase XIV	CA14	Q9ULX7	Lyase	0	1 / 0	CA14	No
Epidermal growth factor receptor erbB1	EGFR	P00533	Kinase	0	1 / 0	EGFR	No
Nischarin	NISCH	Q9Y2I1	Other cytosolic protein	0	1 / 0	NISCH	No
Cytochrome P450 2A6	CYP2A6	P11509	Cytochrome P450	0	1 / 0	CYP2A6	No
Carbonic anhydrase IX	CA9	Q16790	Lyase	0	4 / 2	CA9	No
Cytochrome P450 19A1	CYP19A1	P11511	Cytochrome P450	0	1 / 0	CYP19A1	No
Thymidylate synthase	TYMS	P04818	Transferase	0	1 / 0	TYMS	No
Glycogen synthase kinase-3 beta	GSK3B	P49841	Kinase	0	2 / 0	GSK3B	No
Acetylcholinesterase	ACHE	P22303	Hydrolase	0	1 / 0	ACHE	No
Leukocyte elastase	ELANE	P08246	Protease	0	1 / 0	ELANE	No
Carbonic anhydrase I	CA1	P00915	Lyase	0	3 / 1	CA1	No
